# The effect of cognitive function heterogeneity on depression risk in older adults: a stratified analysis based on functional status

**DOI:** 10.3389/fpubh.2025.1624599

**Published:** 2025-08-18

**Authors:** Taotao Zhang, Shang Li, Zhichen Zhu, Kexin Sha, Yuwen Liu

**Affiliations:** ^1^College of Nursing, Bengbu Medical University, Bengbu, Anhui, China; ^2^College of Health Management, Bengbu Medical University, Bengbu, Anhui, China

**Keywords:** depression, cognitive function, latent profile analysis, functional status, CHARLS

## Abstract

**Backgrounds:**

The mechanisms linking depression and cognitive decline in older adults in the context of global aging are unclear, and functional status may modulate the relationship. This study aimed to reveal the heterogeneity of cognitive functioning in older adults under different physical functional states through latent profile analysis (LPA) and to explore the patterns associated with depressive symptoms.

**Methods:**

Based on the China Health and Retirement Longitudinal Study (CHARLS) 2020 data, 4,158 older adults ≥60 years old were included, and the subtypes of cognitive functions (immediate memory, delayed memory, calculative ability, orientation, and visual construction) were classified by LPA. The associations between different cognitive categories and depressive symptoms were analyzed by stepwise logistic regression. The samples were stratified according to the physical functioning status into “functional intactness “and “functional impairment.”

**Results:**

In the functional intactness group, LPA identified three cognitive profiles, and the risk of depression was significantly higher in the low cognitive function with severe calculative impairment group (19.1%) (OR = 1.52, 95% CI: 1.21–1.91); in the functional impairment group, LPA identified four cognitive profiles, and the risk of depression in the low cognitive function with severe calculative impairment group (21.3%) was 3.37 times higher than that in the high cognitive function group (95% CI: 2.40–4.74), and the low cognitive function with impaired calculative ability group was independently associated with depression risk (OR = 2.65, 95% CI: 1.77–3.94). The strength of the association between low cognition and depression was significantly higher in the functionally impaired population than in the functionally intact population (B-value: 1.25 vs. 0.42, both *p* < 0.001).

**Conclusion:**

Cognitive function heterogeneity significantly affects depression risk through functional status stratification, impaired functioning exacerbates the predictive role of low cognitive functioning for depression, and calculative impairment may be an early marker of executive function impairment. The findings provide a basis for the precise identification of people at high risk of depression and the development of stratified intervention strategies.

## 1 Introduction

As the process of population aging continues to accelerate in China, the mental health of older adults is increasingly becoming a focal issue in public health (1). As a common mental disorder among older adults, the prevalence of depression reaches 10–15% in the community and up to 30% or more in nursing facilities and homes (2, 3). While current antidepressant treatments can alleviate symptoms, challenges such as insufficient efficacy and relapse are particularly prevalent in older adults. For this group, the cumulative rate of relapse following recovery from major depressive symptoms can be as high as 380%, posing a significant threat to both their physical and mental health (4). Studies have shown that geriatric depression is associated with cognitive decline and difficulty functioning in daily physical activities (5–7).

Cognitive functioning is the basis of human higher mental activities, covering multiple core dimensions such as learning, memory, and thinking, which interact dynamically and jointly support individuals’ behavioral decision-making, knowledge construction, and social adaptation (8, 9). Despite the prevalence of physiologic cognitive decline in old age, there is group heterogeneity in the rate and degree of decline among individuals. However, previous studies have mainly used a single scale to assess the presence of cognitive impairment and to explore its association with depressive symptoms, thus neglecting the heterogeneity of cognitive function. As an individual-centered statistical analysis method, Latent Profile Analysis (LPA) can determine the potential categories of research subjects based on their episodic continuous variables and then explore the characteristics and influencing factors of different categories of people. Therefore, considering cognitive function as a profile construct and identifying subgroups with similar cognitive characteristics through LPA provides new perspectives for a deeper understanding of the relationship between geriatric depression and cognitive function.

Although a large number of studies have revealed bidirectional associations between cognitive functioning and depressive symptoms (10–13), the strength and direction of the relationship may vary across different groups of older adults, and this variation may be closely related to the individual’s physical functional status. Older adults with intact functioning may maintain cognitive functioning through social engagement, thus weakening the impact of negative affect caused by cognitive decline. In contrast, individuals with impaired functioning are more likely to have cognitive deficits that translate into frustration with daily tasks due to limitations in their activities, which can lead to an increase in adverse psychological effects (14). However, most of the existing studies consider functional status as a confounding factor rather than a basis for stratified analysis, leading to under-explored mechanisms unique to at-risk populations, such as functionally impaired individuals. Furthermore, although diverse depression intervention strategies exist [e.g., cognitive behavioral therapy (15, 16), physical function rehabilitation (17, 18), community screening programs (19)], evidence for stratified interventions targeting cognitively heterogeneous subgroups remains limited. Clarifying the association patterns between cognitive subtypes and depression under distinct functional statuses could provide critical targets for developing precision interventions.

In this study, we aimed to distinguish between “functional intactness” and “functional impairment” populations, utilize latent profile analysis to reveal the heterogeneity of cognitive functioning in older adults with different physical functioning states, and explore its association with depressive symptoms. Through this study, we hope to provide a basis for the accurate identification of high-risk groups for depression and the development of stratified intervention strategies, thereby promoting the development of research and practice in the field of geriatric mental health.

## 2 Methods

### 2.1 Data sources

This study used data from the fifth wave of the national follow-up survey of the China Health and Retirement Longitudinal Study (CHARLS) 2020. CHARLS is a nationally representative longitudinal cohort study hosted by the National School of Development at Peking University, covering 28 provinces/autonomous regions in China. It employs a multi-stage stratified probability sampling method, and the sample represents socioeconomic and health characteristics (20). The survey has been approved by the Peking University Institutional Review Board (IRB00001052-11015), and all participants signed informed consent forms. This study follows the Helsinki Declaration’s principles and was conducted per established standards and regulations. We included participants aged 60 and above, excluded those with missing or abnormal values for key variables such as cognitive function, depression, physical function, and demographics, and ultimately obtained 4,158 valid samples.

### 2.2 Definition of variables

#### 2.2.1 Cognitive functions

Cognitive functioning was assessed in the CHARLS database by interviewing older adults, with two main components: situational memory and mental state (21–23). Situational memory consists of two dimensions: immediate memory and delayed recall. Participants were asked to memorize 10 semantically unrelated words. After completing the immediate free retelling test, a short delayed recall test was conducted after a few minutes, with one point for each correctly recalled word and 10 points for each immediate and delayed recall. Mental status includes calculative ability, orientation, and visual construction. Respondents’ numerical calculative ability was assessed by asking them to complete five consecutive arithmetic tasks of decreasing from 100 to 7, with 1 point for correct result, totaling 5 points; orientation examined respondents’ ability to correctly recognize the current year, season, month, day and day of the week, with 1 point for each question, totaling 5 points. The visuospatial construction test required the respondents to reproduce the graphs according to the given graphs (24, 25), with 1 point in total 1 point. The psychometric properties of this cognitive function assessment method have been validated by Meng et al. in the CHARLS study (26–28).

#### 2.2.2 Depressed state

In this study, we assessed the depressive state of older adults using the CESD-10 Depression Scale, which has good reliability (Cronbach’s alpha coefficient of 0.795) among Chinese older adults (29, 30). The scale consists of 10 entries, including two positive and eight negative entries, and respondents choose one of the four options that most closely matches their state in the most recent week. For the eight negative entries, answers were scored 0–3 in order according to (1) little or no time (< 1 day); (2) some or a few times (1–2 days); (3) occasionally or moderately (3–4 days); and (4) most or all of the time (5–7 days). Two positive entries were scored in reverse. Scores for all 10 entries were summed for a total score ranging from 0 to 30, with scores ≥10 considered the presence of depressive symptoms (31).

#### 2.2.3 Functional state

Impaired functioning is defined as the need for any assistance with any of the basic activities of daily living (BADL) or instrumental activities of daily living (IADL) (32). BADL include feeding, bathing, dressing, toileting, transferring and continence; IADL include shopping, cooking, housekeeping, managing finances, using the telephone and handling medications, reflecting independence in more complex daily tasks (33). Respondents were considered to be functionally impaired if they reported “difficulty needing help” or “total inability” in any of these activities (34).

#### 2.2.4 Other variables

Covariates were identified through a literature review, and the following covariates were included to take into account confounding factors that may affect cognitive functioning and depressive symptoms: (1) demographic variables: age (continuous variable), gender (male/female), marital status (married/other), and residence (urban/rural); (2) health and lifestyle-related variables: sleep duration (continuous variable), self-rated health (good/fair/poor), number of chronic diseases (0, 1, 2, > = 3), smoking (yes/no), drinking alcohol (yes/no), and participation in social activities (yes/no); and (3) socio-economic variables: level of education (illiterate/primary/secondary/high school and above).

### 2.3 Statistical methods

#### 2.3.1 Descriptive statistics

The basic characteristics of the study sample were first analyzed descriptively. The mean ± standard deviation (Mean ± SD) was used to describe continuous variables; categorical variables were expressed as frequencies (percentages). The study sample was categorized into functional intactness and functional impairment groups based on the physical functional status of the older adults, and comparisons between the groups were made. The *χ*^2^ test (Chi-square test) was used for categorical variables to detect distributional differences between the functional status groups. The Wilcoxon rank-sum test was used for continuous variables to test for significant differences in cognitive functioning between the functional status groups.

#### 2.3.2 LPA

In order to explore the heterogeneity of cognitive functioning in the older adult population, this study used latent profile analysis (LPA) to identify potential categories with similar cognitive functioning based on five dimensions of cognitive functioning: immediate memory, delayed memory, calculative ability, orientation and visuospatial ability (24).

Starting from a single-category model (*k* = 1), the number of categories was gradually increased to a five-category model (*k* = 5), and the optimal profile model was selected based on the fitting metrics. The model evaluation metrics include Akaike Information Criterion (AIC) (35), Bayesian Information Criterion (BIC), Lo-Mendell-Rubin Likelihood Ratio Test (LMR-LRT) and Vuong-Lo-Mendell-Rubin Test (VLMR) (36). In this case, smaller values of AIC and BIC indicate better model fit, and the LMR-LRT and VLMR tests *p* < 0.05 indicate that adding categories significantly improves model fit. Combining the sample proportions of the potential categories and the interpretability of the categories, the optimal classification model was ultimately determined in a comprehensive manner by preventing extreme category groupings with small sample sizes while ensuring that the cognitive function classification had clear clinical explanatory value (37). All LPA analyses were done in Mplus 8.0 software.

#### 2.3.3 Regression

In this study, after identifying different potential categories of cognitive functioning, the relationship between them and depressive symptoms was further explored. Logistic regression analysis was used to assess the association between depressive symptoms as the dependent variable and potential categories of cognitive functioning as the independent variable, and stepwise regression was used to adjust the covariates step by step in order to observe the changes in regression coefficients before and after adjustment. Regression results were expressed as regression coefficients (B), odds ratios (OR), and 95% confidence intervals (95% CI), and the significance level threshold for the two-sided test was set at *p* < 0.05. All statistical analyses were done in Stata 17.0 and SPSS 27.

## 3 Results

### 3.1 Sample characteristics

A total of 4,158 subjects were finally included in this study. The percentage of participants with impaired function was 26.07% (*n* = 1,084), and 73.93% (*n* = 3,074) were functionally intact. The mean (± SD) age was 68.46 ± 5.94 years and included 2,469 (59.38%) male and 1,689 (40.62%) female participants. The mean (± SD) sleep duration of the subjects was 6.05 ± 1.71 h, with the majority of the older adults being of married status (85.3%). More than 85.26% of the older adults suffered from at least one chronic disease, and 43.96% of the older participants suffered from three or more chronic diseases. In addition, the study found that the prevalence of depression in the older adult population was 29.34%, with the prevalence of depression in the functionally intact older adults 22.51%. However, the prevalence of depression in the functionally impaired older adults was as high as 48.71% (see Table 1 for details).

**Table 1:** Descriptive statistics.

Characteristics	Overall (%)	Functional impairment					Functional intactness			
		LCF-SCI	MCF	LCF-ICA	HCF	*p*	LCF-SCI	MCF-ICA	HCF	*p*
Number, %	4,158 (100.0)	231 (21.31)	187 (17.25)	140 (12.92)	526 (48.52)		1,812 (58.95)	674 (21.93)	588 (19.13)	
Age, Mean± SD	68.5 ± 5.9	68.9 ± 6.	69.9 ± 6.3	70.1 ± 6.9	69.4 ± 5.9	0.221	67.9 ± 5.6	68.2 ± 5.8	68.4 ± 6.1	0.133
Gender, %						**0.011**				0.087
Male	2,469 (59.38)	97 (41.99)	90 (48.13)	66 (47.14)	289 (54.94)		647 (35.71)	268 (39.76)	232 (39.46)	
Female	1,689 (40.62)	134 (58.01)	97 (51.87)	74 (52.86)	237 (45.06)		1,165 (64.29)	406 (60.24)	356 (60.54)	
Sleep, Mean ±SD	6.0 ± 1.7	5.5 ± 1.9	5.5 ± 1.8	5.5 ± 1.8	5.5 ± 1.8	0.994	6.2 ± 1.6	6.2 ± 1.6	6.1 ± 1.6	0.302
Marital status						0.419				0.141
Married	3,545 (85.26)	186 (80.52)	154 (82.35)	112 (80.00)	445 (84.60)		1,575 (86.92)	581 (86.20)	492 (83.67)	
Else	613 (14.74)	45 (19.48)	33 (17.65)	28 (20.00)	81 (15.40)		237 (13.08)	93 (13.80)	96 (16.33)	
Residence						0.376				**0.007**
Urban	2,197 (52.84)	106 (45.89)	76 (40.64)	67 (47.86)	252 (47.91)		1,028 (56.73)	336 (49.85)	332 (56.46)	
Rural	1,961 (47.16)	125 (54.11)	111 (59.36)	73 (52.14)	274 (52.09)		784 (43.27)	338 (50.15)	256 (43.54)	
Self-rated health						0.673				0.103
Poor	916 (22.03)	115 (49.78)	85 (45.45)	71 (50.71)	244 (46.39)		246 (13.58)	89 (13.20)	66 (11.22)	
Fair	2,274 (54.69)	99 (42.86)	87 (46.52)	63 (45.00)	251 (47.72)		1,024 (56.51)	381 (56.53)	369 (62.76)	
Good	968 (23.28)	17 (7.36)	15 (8.02)	6 (4.29)	31 (5.89)		542 (29.91)	204 (30.27)	153 (26.02)	
Drinking						**0.031**				0.191
No	2,459 (59.14)	151 (65.37)	131 (70.05)	101 (72.14)	321 (61.03)		1,010 (55.74)	399 (59.20)	346 (58.84)	
Yes	1,699 (40.86)	80 (34.63)	56 (29.95)	39 (27.86)	205 (38.97)		802 (44.26)	275 (40.80)	242 (41.16)	
Smoking						0.839				0.077
No	2,966 (71.33)	175 (75.76)	137 (73.26)	108 (77.14)	401 (76.24)		1,289 (71.14)	448 (66.47)	408 (69.39)	
Yes	1,192 (28.67)	56 (24.24)	50 (26.74)	32 (22.86)	125 (23.76)		523 (28.86)	226 (33.53)	180 (30.61)	
Education						**0.017**				**<0.001**
Illiterate	1,079 (25.95)	94 (40.69)	61 (32.62)	48 (34.29)	163 (30.99)		336 (18.54)	191 (28.34)	186 (31.63)	
Primary school	1,088 (26.17)	60 (25.97)	61 (32.62)	36 (25.71)	128 (24.33)		455 (25.11)	184 (27.30)	164 (27.89)	
Middle school	1,101 (26.48)	47 (20.35)	45 (24.06)	27 (19.29)	141 (26.81)		524 (28.92)	165 (24.48)	152 (25.85)	
High school and above	890 (21.40)	30 (12.99)	20 (10.70)	29 (20.71)	94 (17.87)		497 (27.43)	134 (19.88)	86 (14.63)	
Social activity						0.490				0.400
No	1,955 (47.02)	118 (51.08)	97 (51.87)	71 (50.71)	245 (46.58)		824 (45.47)	327 (48.52)	273 (46.43)	
Yes	2,203 (52.98)	113 (48.92)	90 (48.13)	69 (49.29)	281 (53.42)		988 (54.53)	347 (51.48)	315 (53.57)	
Number of chronic diseases						0.554				0.455
0	595 (14.31)	10 (4.33)	11 (5.88)	8 (5.71)	32 (6.08)		307 (16.94)	116 (17.21)	111 (18.88)	
1	845 (20.32)	27 (11.69)	17 (9.09)	24 (17.14)	68 (12.93)		429 (23.68)	160 (23.74)	120 (20.41)	
2	890 (21.40)	44 (19.05)	39 (20.86)	20 (14.29)	88 (16.73)		423 (23.34)	139 (20.62)	137 (23.30)	
≥3	1,828 (43.96)	150 (64.94)	120 (64.17)	88 (62.86)	338 (64.26)		653 (36.04)	259 (38.43)	220 (37.41)	
Depression						**<0.001**				**<0.001**
No	2,938 (70.66)	73 (31.60)	116 (62.03)	52 (37.14)	315 (59.89)		1,452 (80.13)	503 (74.63)	427 (72.62)	
Yes	1,220 (29.34)	158 (68.40)	71 (37.97)	88 (62.86)	211 (40.11)		360 (19.87)	171 (25.37)	161 (27.38)	

Functional impairment, LCF-SCI, Low Cognitive Function with Severe Calculative Impairment. MCF, Moderate Cognitive Function Group; LCF-ICA, Low Cognitive Function with Impaired Calculative Ability. HCF, High Cognitive Function Group; Functional intactness, LCF-SCI, Low Cognitive Function with Severe Calculative Impairment; MCF-ICA, Moderate Cognitive Function with Impaired Calculative Ability; HCF, High Cognitive Function Group. Boldface indicates statistical significance (*p* < 0.05).

### 3.2 Potential profile analysis

**Figure 1:**
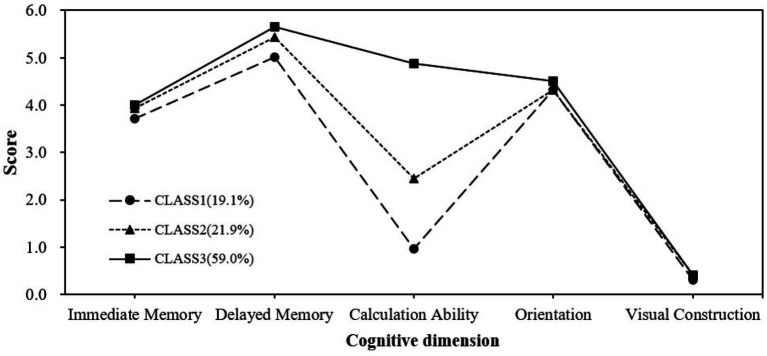
Proportional distribution of functional intactness subgroups.

Tables 2, 3 show the LPA model fit indices for cognitive function in stratified populations. In the fully functional participants, the model fit increased as the potential subgroups increased with progressively higher AIC, BIC, and aBIC values. Entropy was highest at category 3, then decreased slightly at category 4, and rose again to 1 at category 5, all exceeding 0.8, indicating good distinguishability and reliable categorization. The *p*-values for LMRT and BLRT from categories 1–5 are all less than 0.05, indicating that each added category significantly improves the model. However, both categories 4 and 5 have a minimum category share of < 5%, according to the theoretical significance and practical interpretability of the model emphasized by Nylund et al. (36), too few categories (< 5%) may lack relevance and stability, and retaining such categories may lead to overfitting of the model, so categories 4 and 5 are excluded, and the minimum category share of 19.13% in the model of category 3 is more in line with the statistical robustness requirements. Finally, we chose the 3-category model as the best model. Figure 1 represents the sample proportion and estimated probability of cognitive categorization in the functional intactness group, and based on the difference of the dimensions in the figure, we named the category 1 (19.1% of the total) as the group of low cognitive function with severe calculative impairment (LCF-SCI); the category 2 (21.9%) as the group of moderate cognitive function with impaired calculative ability (MCF-ICA); and the category 3 (59.0% of the total) was good in all dimensions and named the high cognitive function group (HCF).

**Table 2:** Functional intactness group: latent profile analysis (LPA) indicators.

Classes	AIC	BIC	ABIC	LMR	BLRT	Entropy	Class probability
1	48190.681	48250.988	48219.214	–	–	–	–
2	45432.399	45589.198	45506.585	<0.001	<0.001	0.925	40.73/59.27
3	44064.796	44257.779	44156.102	<0.001	<0.001	0.984	19.13/21.93/58.95
4	43921.327	44150.495	44029.754	<0.001	<0.001	0.971	19.13/1.24/21.93/57.71
5	42754.394	43019.746	42879.941	0.004	<0.001	1	1.17/17.96/51.85/12.23/16.79

AIC, Akaike information criterion; BIC, Bayesian information criterion; ABIC, sample size adjusted BIC; LMR, Lo-Mendell-Rubin likelihood radio test; BLRT, Bootstrap likelihood ratio test.

**Table 3:** Functional impairment group: latent profile analysis (LPA) indicators.

classes	AIC	BIC	ABIC	LMR	BLRT	Entropy	Class probability
1	16904.79	17004.558	16941.034	–	–	–	–
2	16241.035	16370.733	16288.152	<0.001	<0.001	0.921	44.09/55.91
3	15755.066	15914.695	15813.056	<0.001	<0.001	0.983	23.54/21.67/54.79
4	15408.212	15597.772	15477.076	<0.001	<0.001	0.994	21.31/ 17.25/ 48.52/12.92
5	14426.774	14646.264	14506.511	0.158	<0.001	1	21.31/12.92/10.98/48.52/6.27

AIC, Akaike information criterion; BIC, Bayesian information criterion; ABIC, sample size adjusted BIC; LMR, Lo–Mendell–Rubin likelihood radio test; BLRT, Bootstrap likelihood ratio test.

**Figure 2:**
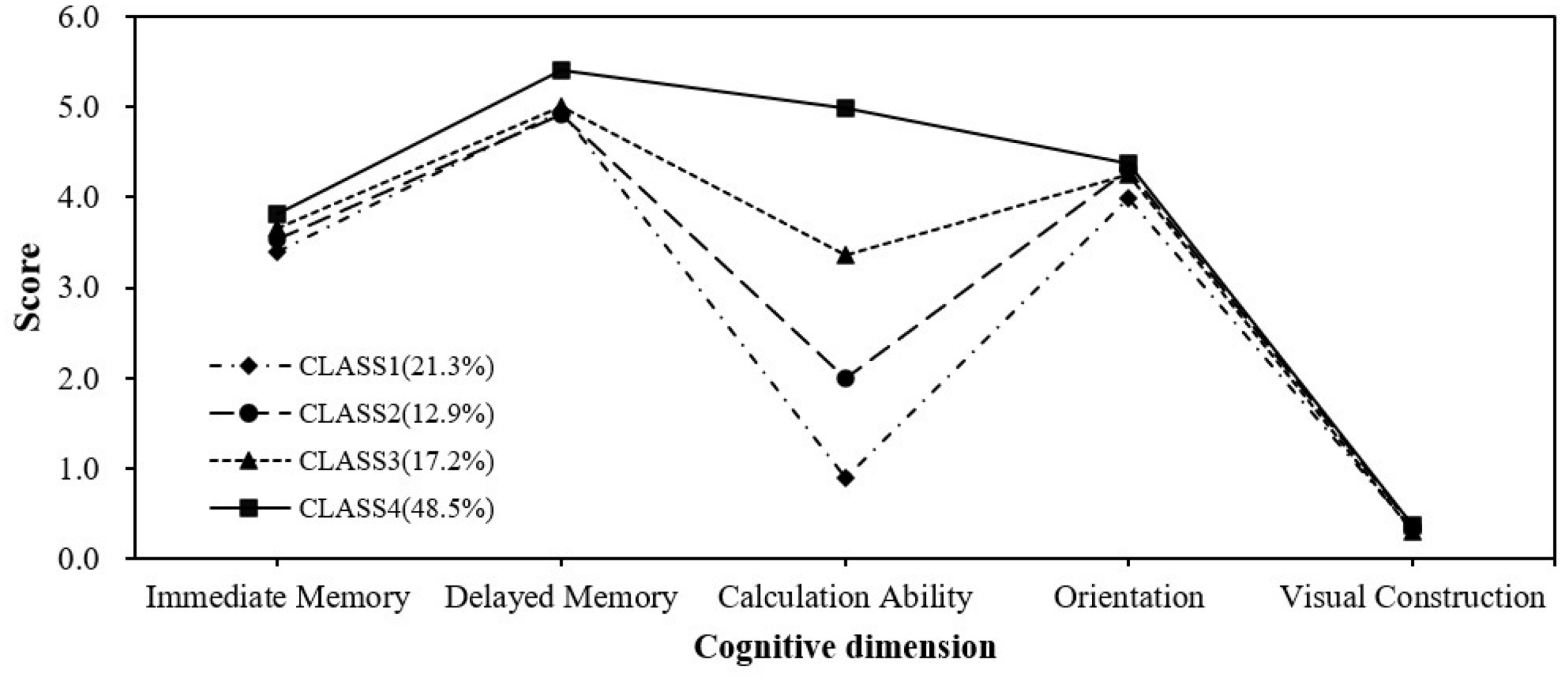
Proportional distribution of functional impairment subgroups.

For functionally impaired participants, the *p*-value for category 4 was < 0.05, whereas the *p*-value for category 5 was > 0.05, indicating no significant improvement from category 4 to 5. The entropy value for category 4 was 0.994, indicating a high degree of clarity in the classification of the categories. Category 4 achieved an optimal balance between model fit, statistical significance, and practical explanatory properties. Finally, we chose the 4-category model for analysis. Figure 2 represents the categorization results of the functionally impaired population. Category 1 (21.3% of the population) has overall low cognitive ability and severely impaired numeracy, so the first category is named the low cognitive function with severe calculative impairment group (LCF-SCI); In category 2 (12.9% of the percentage), other dimensions were better and calculative ability was poor, so category 2 was named the low cognitive function with impaired calculative ability group (LCF-ICA); In category 3 (17.2% share) named moderate cognitive functioning group (MCF). In category 4 (48.5% of the percentage), cognitive ability is generally higher than in other cognitive categories, so category 4 is named the high cognitive function group (HCF).

### 3.3 Depression risk analysis

**Figure 3:**
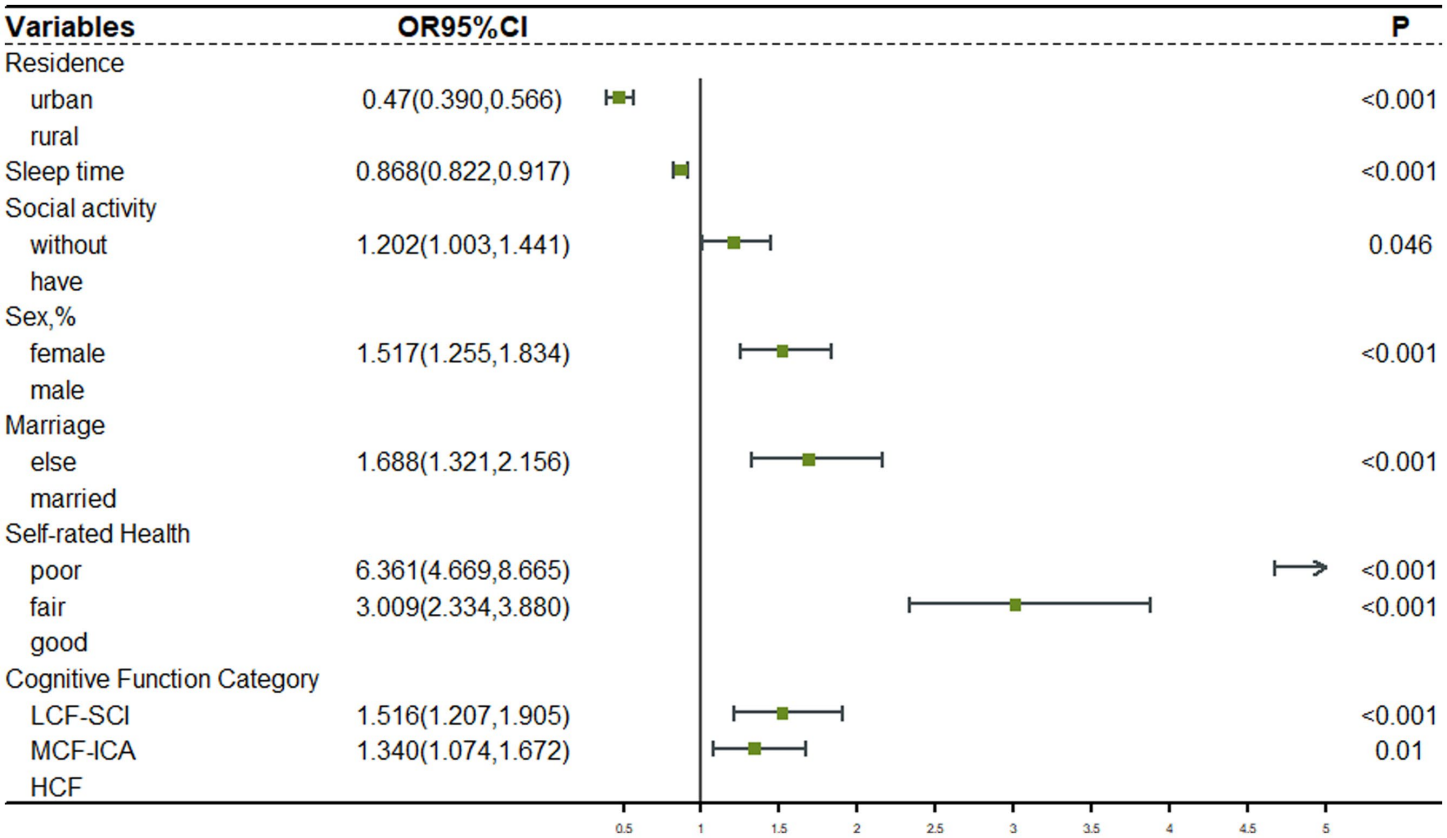
Logistic regression analysis of depression in the functional intactness group. LCF-SCI, Low Cognitive Function with Severe Calculative Impairment; MCF-ICA, Moderate Cognitive Function with Impaired Calculative Ability; HCF, High Cognitive Function Group.

**Figure 4:**
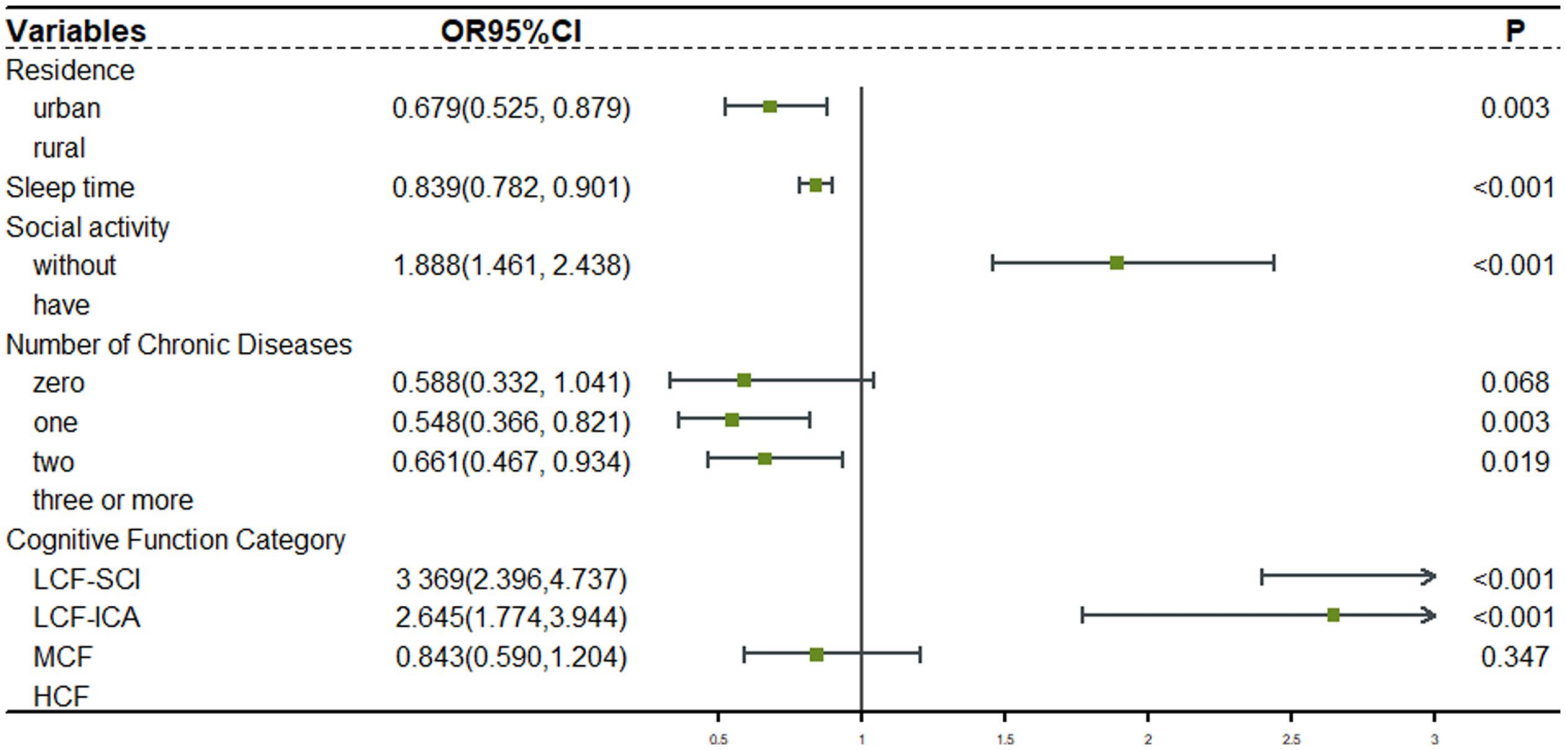
Logistic regression analysis of depression in the functional impairment group. LCF-SCI, Low Cognitive Function with Severe Calculative Impairment. MCF, Moderate Cognitive Function Group; LCF-ICA, Low Cognitive Function with Impaired Calculative Ability. HCF, High Cognitive Function Group.

Forward stepwise logistic regression was used to analyze the relationship between depression and influential factors. For older participants with intact functioning, after adjusting for gender, marriage, residence, sleep duration, social activities, and self-rated health, and using the high cognitive functioning group as a control group, Figure 3 results showed that the low cognitive functioning with severe calculative impairment group (OR = 1.516, 95% CI: 1.207–1.905) and the moderate cognitive function with impaired calculative ability group (OR = 1.340, 95% CI: 1.074–1.672) were associated with a higher risk of depression. For older participants with impaired functioning, after adjusting for residence, sleep duration, social activities, and number of chronic diseases, and using the high cognitive functioning group as a control group, Figure 4 results showed that there was no statistically significant difference in the risk of depression in the moderate cognitive function group (OR = 0.843, 95% CI: 0.590–1.204) but impaired computational functioning showed a significant risk gradient, with low cognitive functioning with severe calculative impairment group showed a more than 2.3-fold increase in depression risk (OR = 3.369, 95% CI: 2.396–4.737), and the low cognitive function with impaired calculative ability group also showed a 1.6-fold increase in risk (OR = 2.645, 95% CI: 1.774–3.944).

## 4 Discussion

In this study, we revealed the heterogeneous characteristics of cognitive functioning in older adults with different physical functioning states employing latent profile analysis (LPA) and delved into the association between cognitive profiles and depressive symptoms. The results showed that the cognitive functions of functionally intact and functionally impaired older adults showed significantly different stratification structures, with the strength of the association between the low cognitive function group and depressive symptoms being particularly prominent (B-values of 0.416 and 1.251, respectively, both *p* < 0.001). This finding not only confirms the association between cognitive function and depressive symptoms in previous studies but also provides a new perspective for understanding the mechanism of action of the two through the fine delineation of cognitive subtypes.

### 4.1 Multidimensional analysis of heterogeneity in cognitive functioning

Among functionally intact older adults, the LPA model identified three types of cognitive profiles, with differences centered on calculative ability and delayed memory. The low cognitive functioning group was significantly lower than the other groups on these two measures. In contrast, no significant between-group differences existed on the immediate memory, orientation, and visual construction dimensions. This result supports the “asynchronous decline model of cognitive capacity,” i.e., the temporal heterogeneity of functional loss in different cognitive domains (38). The early decline of computational power, a prefrontal-parietal network-dependent higher cognitive function (39), may serve as a sensitive marker of cognitive reserve depletion (5).

Cognitive stratification of functionally impaired older adults was more complex, and the separate division of the four types of profiles into the severely impaired low cognitive function with severe calculative impairment group was clinically significant. This group did not differ significantly from the others in immediate memory and visual construction dimensions. However, it had significantly calculative impairment and a risk of depression that was 3.369 times higher than that of the high cognitive functioning group. This finding suggests that numeracy deficits may be a “window indicator” of executive function impairment (40). Decreased physical activity triggers computational decline by disrupting the efficiency of prefrontal lobe regulation (41), forming a pathological chain of “impaired function → executive impairment → computational deficits → risk of depression,” which is consistent with the findings of Zhu L et al. (42).

### 4.2 Neurobiological mechanisms of cognitive-depressive associations

The present study found a significantly higher risk of depression in the low cognitive functioning group (adjusted OR = 1.52, 95% CI: 1.21–1.91 in the functional intactness group and adjusted OR = 3.37, 95% CI: 2.43–4.67 in the functional impairment group), which was closely related to prefrontal cortex hypofunction (43). Structural damage to the prefrontal-limbic system-hippocampal loop is more likely to exacerbate feelings of low self-esteem and helplessness through negative cognitive biases (44–47). The present study further suggests that computational power may be a more direct characterization of the functional status of this loop.

Animal experimental evidence that exercise promotes hippocampal neurogenesis by upregulating brain-derived neurotrophic factor (BDNF) levels (48) suggests that the cognitive advantage in the functionally intact group may be related to a protective mechanism of exercise-related neuroplasticity. Future studies could test this hypothesis using serum BDNF assays and cranial MRI scans in human samples.

### 4.3 Methodological innovations and clinical insights

In contrast to traditional studies that consider cognitive function as a single variable, this study used the LPA model to reveal the heterogeneity of cognitive subtypes and analyze the cognitive-depressive associations in older adults with different functional statuses. Based on the identified population heterogeneity, we propose stratified intervention strategies: For the functional intactness group, primary interventions should focus on calculative ability training (e.g., arithmetic-based games) and social engagement. For the functional impairment group, multimodal interventions integrating physical rehabilitation and executive function training are recommended.

Notably, our findings highlight calculative impairment as a significant predictor of depression risk, suggesting its potential as an early biomarker for screening. We advocate incorporating brief calculative tests (e.g., adopting CHARLS-style arithmetic tasks) into community-based depression screening systems for older adults. This cost-effective, easily administrable tool could facilitate preliminary identification of high-risk individuals. Such a tiered intervention model not only optimizes resource allocation but also enables precision prevention and treatment tailored to individuals’ functional statuses.

### 4.4 Research limitations and future directions

This study provides new perspectives on the mechanisms of cognition-depression interactions, but it is only a preliminary exploration. Several limitations remain: (1) The study’s cross-sectional design limits causal inferences and requires longitudinal follow-up in the future. (2) Due to data limitations, we did not assess the association between other dimensions of cognitive functioning and depression, such as “language functioning.” (3) The data for this study were collected during the COVID-19 pandemic, and the control of the pandemic may have led to a systematic bias in the scores of the Self-Rating Scale of Depressive Symptoms in the older adults. Therefore, when extrapolating the depressive symptom-cognitive functioning association model constructed in this study to regular social situations, structural differences in environmental stressors need to be carefully considered. (4) The gender ratio of the study sample was unbalanced, and future studies need to include more balanced samples to improve the generalizability of this study. Future multicenter and longitudinal studies are expected to validate the model further and promote the development of precise intervention strategies.

## 5 Conclusion

In summary, the present study reveals the differential impact of cognitive heterogeneity on depression risk through an in-depth profiling of cognitive function in older adults with different functional statuses. In the group of functional intactness older adults, we identified three patterns of cognitive functioning, in which individuals with low cognitive functioning had a significantly elevated risk of depression. In contrast, among the four cognitive subtypes in functionally impaired older adults, the group with low cognitive function with severe calculative impairment in particular had the most prominent risk of depression. Notably, the impaired functioning status not only exacerbated the strength of the association between low cognitive functioning and depression but also highlighted the early warning value of numeracy as a core indicator of executive functioning in the development of depression. Based on population heterogeneity, we propose stratified intervention strategies: the functional intactness group should receive primary interventions focusing on calculative ability training (e.g., arithmetic-based games) and social engagement, while the functionally impaired group requires multimodal interventions integrating physical rehabilitation and executive function training. We recommend incorporating brief calculative tests (e.g., CHARLS’s arithmetic tasks) into community-based depression screening systems to enable precise identification of high-risk individuals. As this study is limited by its cross-sectional design, future longitudinal research is needed to verify the temporal relationship between cognitive decline and depression, thereby providing more robust theoretical foundations for precision mental health interventions in older adults.

## Data Availability

Publicly available datasets were analyzed in this study. This data can be found at: https://charls.pku.edu.cn.

## References

[1] Fang EF Scheibye-Knudsen M Jahn HJ Li J Ling L Guo H. A research agenda for aging in China in the 21st century. Ageing Res Rev. (2015) 24:197–205. doi: 10.1016/j.arr.2015.08.003, PMID: 26304837 PMC5179143

[2] Birrer RB Vemuri SP. Depression in later life: a diagnostic and therapeutic challenge. Am Fam Physician. (2004) 69:2375–82. 15168957

[3] Leon FG Ashton AK D’Mello DA Dantz B Hefner J Matson GA. Depression and comorbid medical illness: therapeutic and diagnostic challenges. J Fam Pract. (2003):S19–33. 14693075

[4] Mueller TI Leon AC Keller MB Solomon DA Endicott J Coryell W. Recurrence after recovery from major depressive disorder during 15 years of observational follow-up. Am J Psychiatry. (1999) 156:1000–6. doi: 10.1176/ajp.156.7.1000, PMID: 10401442

[5] Almeida OP. Prevention of depression in older age. Maturitas. (2014) 79:136–41. doi: 10.1016/j.maturitas.2014.03.005, PMID: 24713453

[6] Maier A Riedel-Heller SG Pabst A Luppa M. Risk factors and protective factors of depression in older people 65+. A systematic review. PLoS One. (2021) 16:e0251326. doi: 10.1371/journal.pone.0251326, PMID: 33983995 PMC8118343

[7] Shimada H Park H Makizako H Doi T Lee S Suzuki T. Depressive symptoms and cognitive performance in older adults. J Psychiatr Res. (2014) 57:149–56. doi: 10.1016/j.jpsychires.2014.06.004, PMID: 25023083

[8] Bahar-Fuchs A Martyr A Goh AM Sabates J Clare L. Cognitive training for people with mild to moderate dementia. Cochrane Database Syst Rev. (2019) 3:CD013069. doi: 10.1002/14651858.CD013069.pub2, PMID: 30909318 PMC6433473

[9] Xian G Chai Y Gong Y He W Ma C Zhang X. The relationship between healthy lifestyles and cognitive function in Chinese older adults: the mediating effect of depressive symptoms. BMC Geriatr. (2024) 24:299. doi: 10.1186/s12877-024-04922-5, PMID: 38549104 PMC10979595

[10] Alexopoulos GS Meyers BS Young RC Mattis S Kakuma T. The course of geriatric depression with “reversible dementia”: a controlled study. Am J Psychiatry. (1993) 150:1693–9. doi: 10.1176/ajp.150.11.1693, PMID: 8105707

[11] Guan T Zhang C Zou X Chen C Zhou L Wu X. The influence of alcohol consumption, depressive symptoms and sleep duration on cognition: results from the China health and retirement longitudinal study. Int J Environ Res Public Health. (2022) 19:12574. doi: 10.3390/ijerph191912574, PMID: 36231874 PMC9566793

[12] Krishnan KR McDonald WM Doraiswamy PM Tupler LA Husain M Boyko OB. Neuroanatomical substrates of depression in the elderly. Eur Arch Psychiatry Clin Neurosci. (1993) 243:41–6. doi: 10.1007/BF02191522, PMID: 8399409

[13] Wang G Zhou Y Duan J Kan Q Cheng Z Tang S. Effects of adverse childhood health experiences on cognitive function in Chinese middle-aged and older adults: mediating role of depression. BMC Public Health. (2023) 23:1293. doi: 10.1186/s12889-023-16169-7, PMID: 37407916 PMC10320919

[14] Bozo O Toksabay NE Kürüm O. Activities of daily living, depression, and social support among elderly Turkish people. J Psychol. (2009) 143:193–206. doi: 10.3200/JRLP.143.2.193-206, PMID: 19306681

[15] Cuijpers P Berking M Andersson G Quigley L Kleiboer A Dobson KS. A meta-analysis of cognitive-behavioural therapy for adult depression, alone and in comparison with other treatments. Can J Psychiatr. (2013) 58:376–85. doi: 10.1177/070674371305800702, PMID: 23870719

[16] Gould RL Coulson MC Howard RJ. Cognitive behavioral therapy for depression in older people: a meta-analysis and meta-regression of randomized controlled trials. J Am Geriatr Soc. (2012) 60:1817–30. doi: 10.1111/j.1532-5415.2012.04166.x, PMID: 23003115

[17] Carek PJ Laibstain SE Carek SM. Exercise for the treatment of depression and anxiety. Int J Psychiatry Med. (2011) 41:15–28. doi: 10.2190/PM.41.1.c, PMID: 21495519

[18] Kandola A Ashdown-Franks G Hendrikse J Sabiston CM Stubbs B. Physical activity and depression: towards understanding the antidepressant mechanisms of physical activity. Neurosci Biobehav Rev. (2019) 107:525–39. doi: 10.1016/j.neubiorev.2019.09.040, PMID: 31586447

[19] Ainsworth NJ Marawi T Maslej MM Blumberger DM McAndrews MP Perivolaris A. Cognitive outcomes after antidepressant pharmacotherapy for late-life depression: a systematic review and Meta-analysis. Am J Psychiatry. (2024) 181:234–45. doi: 10.1176/appi.ajp.20230392, PMID: 38321915

[20] Zhao Y Hu Y Smith JP Strauss J Yang G. Cohort profile: the China health and retirement longitudinal study (CHARLS). Int J Epidemiol. (2014) 43:61–8. doi: 10.1093/ije/dys203, PMID: 23243115 PMC3937970

[21] Hu Y Peng W Ren R Wang Y Wang G. Sarcopenia and mild cognitive impairment among elderly adults: the first longitudinal evidence from CHARLS. J Cachexia Sarcopenia Muscle. (2022) 13:2944–52. doi: 10.1002/jcsm.13081, PMID: 36058563 PMC9745544

[22] Xu X Xu Y Shi R. Association between obesity, physical activity, and cognitive decline in Chinese middle and old-aged adults: a mediation analysis. BMC Geriatr. (2024) 24:54. doi: 10.1186/s12877-024-04664-4, PMID: 38212676 PMC10785530

[23] Yao Y Wang K Xiang H. Association between cognitive function and ambient particulate matters in middle-aged and elderly Chinese adults: evidence from the China health and retirement longitudinal study (CHARLS). Sci Total Environ. (2022) 828:154297. doi: 10.1016/j.scitotenv.2022.154297, PMID: 35288137 PMC9112163

[24] Liu Y-H Chen M-T He Y-Y Chen M Liang J-R Jia F-J. Cognitive impairment and depression precede increased HDL-C levels in middle-aged and older Chinese adults: cross-lagged panel analyses. Lipids Health Dis. (2024) 23:288. doi: 10.1186/s12944-024-02285-9, PMID: 39252009 PMC11382475

[25] Ma Y Wu X Zhao Y Hong W Luan Y Song P. Relationships between muscle strength, lung function, and cognitive function in Chinese middle-aged and older adults: a study based on the China health and retirement longitudinal study (CHARLS). J Formos Med Assoc. (2025) 124:171–7. doi: 10.1016/j.jfma.2024.04.001, PMID: 38594163

[26] Meng Q Wang H Strauss J Langa KM Chen X Wang M. Validation of neuropsychological tests for the China health and retirement longitudinal study harmonized cognitive assessment protocol. Int Psychogeriatr. (2019) 31:1709–19. doi: 10.1017/S1041610219000693, PMID: 31309907 PMC8082093

[27] Qin T Yan M Fu Z Song Y Lu W Fu A. Association between anemia and cognitive decline among Chinese middle-aged and elderly: evidence from the China health and retirement longitudinal study. BMC Geriatr. (2019) 19:305. doi: 10.1186/s12877-019-1308-7, PMID: 31718564 PMC6849217

[28] Zheng G Zhou B Fang Z Chen X Liu M He F. Long-term visit-to-visit blood pressure variability and cognitive decline among patients with hypertension: a pooled analysis of 3 National Prospective Cohorts. J Am Heart Assoc. (2024) 13:e035504. doi: 10.1161/JAHA.124.035504, PMID: 38934858 PMC11255695

[29] Chen H Mui AC. Factorial validity of the Center for Epidemiologic Studies Depression Scale short form in older population in China. Int Psychogeriatr. (2014) 26:49–57. doi: 10.1017/S1041610213001701, PMID: 24125553

[30] Guo M Li Z Chen Y Chen X Cheng Z Tang Z. Study on the relationship between depressive symptoms and internet use in the older adults under the background of population aging-evidence based on CHARLS 2018 and 2020. BMC Public Health. (2025) 25:1057. doi: 10.1186/s12889-025-22141-4, PMID: 40108552 PMC11921652

[31] Zheng Y Zhang T Yang S Wang F Zhang L Liu Y. Using machine learning to predict the probability of incident 2-year depression in older adults with chronic diseases: a retrospective cohort study. BMC Psychiatry. (2024) 24:870. doi: 10.1186/s12888-024-06299-6, PMID: 39623372 PMC11610371

[32] Zhang L Cui H Chen Q Li Y Yang C Yang Y. A web-based dynamic nomogram for predicting instrumental activities of daily living disability in older adults: a nationally representative survey in China. BMC Geriatr. (2021) 21:311. doi: 10.1186/s12877-021-02223-9, PMID: 34001030 PMC8127258

[33] Lu X Yao Y Jin Y. Digital exclusion and functional dependence in older people: findings from five longitudinal cohort studies. EClinicalMedicine. (2022) 54:101708. doi: 10.1016/j.eclinm.2022.101708, PMID: 36353265 PMC9637559

[34] Yu Q Li Z Yang C Zhang L Xing M Li W. Predicting functional dependency using machine learning among a middle-aged and older Chinese population. Arch Gerontol Geriatr. (2023) 115:105124. doi: 10.1016/j.archger.2023.105124, PMID: 37454417

[35] Akaike H. A new look at the statistical model identification. IEEE Trans Autom Control. (1974) 19:716–23. doi: 10.1109/TAC.1974.1100705

[36] Nylund KL Asparouhov T Muthé BO. Deciding on the number of classes in latent class analysis and growth mixture modeling: a Monte Carlo simulation study. Struct Equ Modeling. (2007) 14:535–69. doi: 10.1080/10705510701575396

[37] Yang C-C. Evaluating latent class analysis models in qualitative phenotype identification. Comput Stat Data Anal. (2006) 50:1090–104. doi: 10.1016/j.csda.2004.11.004

[38] Salthouse T. Consequences of age-related cognitive declines. Annu Rev Psychol. (2012) 63:201–26. doi: 10.1146/annurev-psych-120710-100328, PMID: 21740223 PMC3632788

[39] Duncan J Owen AM. Common regions of the human frontal lobe recruited by diverse cognitive demands. Trends Neurosci. (2000) 23:475–83. doi: 10.1016/s0166-2236(00)01633-7, PMID: 11006464

[40] Pizzie RG Raman N Kraemer DJM. Math anxiety and executive function: neural influences of task switching on arithmetic processing. Cogn Affect Behav Neurosci. (2020) 20:309–25. doi: 10.3758/s13415-020-00770-z, PMID: 32112298

[41] Ávila R Miotto EC. Funções executivas no envelhecimento normal e na doença de Alzheimer. J Bras Psiquiatr. (2003) 52:53–63.

[42] Zhu L Li L Wang L Jin X Zhang H. Physical activity for executive function and activities of daily living in AD patients: a systematic review and Meta-analysis. Front Psychol. (2020) 11:560461. doi: 10.3389/fpsyg.2020.560461, PMID: 33343442 PMC7744293

[43] Clark DA Beck AT. Cognitive theory and therapy of anxiety and depression: convergence with neurobiological findings. Trends Cogn Sci. (2010) 14:418–24. doi: 10.1016/j.tics.2010.06.007, PMID: 20655801

[44] Disner SG Beevers CG Haigh EAP Beck AT. Neural mechanisms of the cognitive model of depression. Nat Rev Neurosci. (2011) 12:467–77. doi: 10.1038/nrn3027, PMID: 21731066

[45] Mayberg HS. Limbic-cortical dysregulation: a proposed model of depression. J Neuropsychiatry Clin Neurosci. (1997) 9:471–81. doi: 10.1176/jnp.9.3.471, PMID: 9276848

[46] Reyna VF Nelson WL Han PK Dieckmann NF. How numeracy influences risk comprehension and medical decision making. Psychol Bull. (2009) 135:943–73. doi: 10.1037/a0017327, PMID: 19883143 PMC2844786

[47] Yin J John A Cadar D. Bidirectional associations of depressive symptoms and cognitive function over time. JAMA Netw Open. (2024) 7:e2416305. doi: 10.1001/jamanetworkopen.2024.16305, PMID: 38861255 PMC11167501

[48] van Praag H Kempermann G Gage FH. Running increases cell proliferation and neurogenesis in the adult mouse dentate gyrus. Nat Neurosci. (1999) 2:266–70. doi: 10.1038/6368, PMID: 10195220

